# Cervical Total Disc Replacement is Superior to Anterior Cervical Decompression and Fusion: A Meta-Analysis of Prospective Randomized Controlled Trials

**DOI:** 10.1371/journal.pone.0117826

**Published:** 2015-03-30

**Authors:** Yujie Zhang, Chengzhen Liang, Yiqing Tao, Xiaopeng Zhou, Hao Li, Fangcai Li, Qixin Chen

**Affiliations:** Department of Orthopedic Surgery, 2nd Affiliated Hospital, School of Medicine, Zhejiang University, 88 Jie Fang Road, Hangzhou, 310009, Zhejiang, People’s Republic of China; Toronto Western Hospital, CANADA

## Abstract

**Background:**

Despite being considered the standard surgical procedure for symptomatic cervical disc disease, anterior cervical decompression and fusion invariably accelerates adjacent segment degeneration. Cervical total disc replacement is a motion-preserving procedure developed as a substitute to fusion. Whether cervical total disc replacement is superior to fusion remains unclear.

**Methods:**

We comprehensively searched PubMed, EMBASE, Medline, and the Cochrane Library in accordance with the inclusion criteria to identify possible studies. The retrieved results were last updated on December 12, 2014. We classified the studies as short-term and midterm follow-up.

**Results:**

Nineteen randomized controlled trials involving 4516 cases were identified. Compared with anterior cervical decompression and fusion, cervical total disc replacement had better functional outcomes (neck disability index [NDI], NDI success, neurological success, neck pain scores reported on a numerical rating scale [NRS], visual analog scales scores and overall success), greater segmental motion at the index level, fewer adverse events and fewer secondary surgical procedures at the index and adjacent levels in short-term follow-up (P < 0.05). With midterm follow-up, the cervical total disc replacement group indicated superiority in the NDI, neurological success, pain assessment (NRS), and secondary surgical procedures at the index level (P < 0.05). The Short Form 36 (SF-36) and segmental motion at the adjacent level in the short-term follow-up showed no significant difference between the two procedures, as did the secondary surgical procedure rates at the adjacent level with midterm follow-up (P > 0.05).

**Conclusions:**

Cervical total disc replacement presented favorable functional outcomes, fewer adverse events, and fewer secondary surgical procedures. The efficacy and safety of cervical total disc replacement are superior to those of fusion. Longer-term, multicenter studies are required for a better evaluation of the long-term efficacy and safety of the two procedures.

## Introduction

Anterior cervical decompression and fusion (ACDF) is generally considered the standard surgical procedure for cervical myelopathy or radiculopathy with degenerative disc disease [1,2]. ACDF typically consists of decompression, grafting and plate fixation [3]. Compared to other spinal procedures, ACDF demonstrates higher success rates, including more favorable outcomes and relief of symptoms [4]. Complications invariably occur with this procedure. Pseudarthrosis and junctional degeneration, commonly known as adjacent segment disease, are the most notable complications, which is explained by bio-mechanical studies which indicate that adjacent levels of cervical fusion present higher intradiscal pressures and increased segmental motion [5–7]. In recent decades, data have shown that as a consequence of fusion surgery, the incidence of adjacent segment degeneration varies from 3% to 8% annually, and approximately 25% of the patients would present with clinically significant adjacent segment disease within 10 years after the initial surgery [6,8,9].

Cervical total disc replacement (CTDR) is a relatively new motion-preserving procedure that has been regarded as a substitute for ACDF [10–12]. The function of CTDR in motion preservation of the adjacent segment remains controversial. Additionally, the incidence of heterotopic ossification and the effect on adjacent-level disease resulting from CTDR are disputed in clinical studies [13,14]. To address these issues, we collected prospective evidence and performed a meta-analysis to compare the efficacy and safety of CTDR and ACDF for the treatment of symptomatic cervical disc disease.

## Materials and Methods

### Search Strategy and Criteria

Two independent reviewers (YJZ and CZL) systematically searched electronic databases (PubMed, EMBASE, Medline, and the Cochrane Library) with a limit of ‘‘clinical trial”. The retrieved results were last updated on December 12, 2014. We used the following terms and Boolean operators: ‘‘(Anterior cervical decompression and fusion OR anterior cervical arthrodesis OR ACDF OR fusion) AND (Artificial cervical disc replacement OR CTDR OR Cervical arthroplasty OR disc implants OR disc prostheses OR CDA)”. We included studies that met the following criteria: (1) the target patients had symptomatic cervical disc disease and underwent CTDR or ACDF; (2) the patients were older than 18 years; (3) postoperative follow-up extended at least 2 years for the included patients; (4) the outcomes included at least one of the following conditions: 1) neck disability index (NDI); 2) NDI success; 3) neck and arm pain assessments measured by visual analog scales (VAS) or the numerical rating scale (NRS); 4) Short Form 36(SF-36) mental or physical health surveys (physical component summary or mental component summary scores); 5) Neurological status; 6) flexion-extension ROM at the index and adjacent levels; 7) secondary surgical procedures; 8) adverse events; 9) overall success; (5) the trial was a randomized controlled trial (RCT). Trials were excluded according to the following criteria: (1) the articles were observational studies, case reports, or reviews; (2) the outcomes were graphic without numerical values; (3) the same data had been published previously; (4) the RCTs had a follow-up of less than 2 years.

### Data Extraction

For each eligible trial, the elements of the data, including the study design, intervention protocol, sample size, demographic data (age, gender distribution), trial duration, follow-up times, trial outcomes and loss to follow-up, were independently extracted by two reviewers (YJZ and CZL). If any disagreements existed, a third reviewer (YQT) was involved in the discussion until consensus was reached.

### Quality Assessment

Two reviewers (YJZ and CZL) independently evaluated the methodological quality of the included trials in accordance with a 12-item scale recommended by the Cochrane Back Review Group [15]. If at least six of the 12 criteria, including randomization, allocation concealment, blinding (of the patients, assessors, and surgeons), similar baseline, selective reporting, loss to follow-up, patient compliance, similar timing and intention-to-treat (ITT) analysis, were met without serious flaws, the studies were rated as having ‘‘low risk of bias”. Otherwise, the studies were rated as having ‘‘high risk of bias”. Additionally, the GRADE (Grades of Recommendation, Assessment, Development and Evaluation) approach was used to evaluate the strength of evidence [16]. Based on parameters such as study design, precision, directness, consistency and risk of bias, the quality assessment was classified as very low, low, moderate or high.

### Statistical Analysis

The odds ratio (OR) and the corresponding 95% confidence interval (CI) were assessed for the dichotomous outcomes, and the standardized mean difference (SMD) and 95% CI were assessed for the continuous outcomes. The chi-square test and Higgin’s *I* ^2^ test were used to evaluate the heterogeneity. A *p* value less than 0.10 for the chi-square test or *I* ^2^ values exceeding 50% indicated substantial heterogeneity. A fixed-effect model was used if significantly statistical heterogeneity was absent; otherwise, a random-effect model was applied. Because of the limited number of included studies, we did not assess the possibility of publishing bias. We used Review Manager Software (RevMan Version 5.2, The Cochrane Collaboration, Copenhagen, Denmark) to conduct the statistical analysis.

## Results

**Fig 1:**
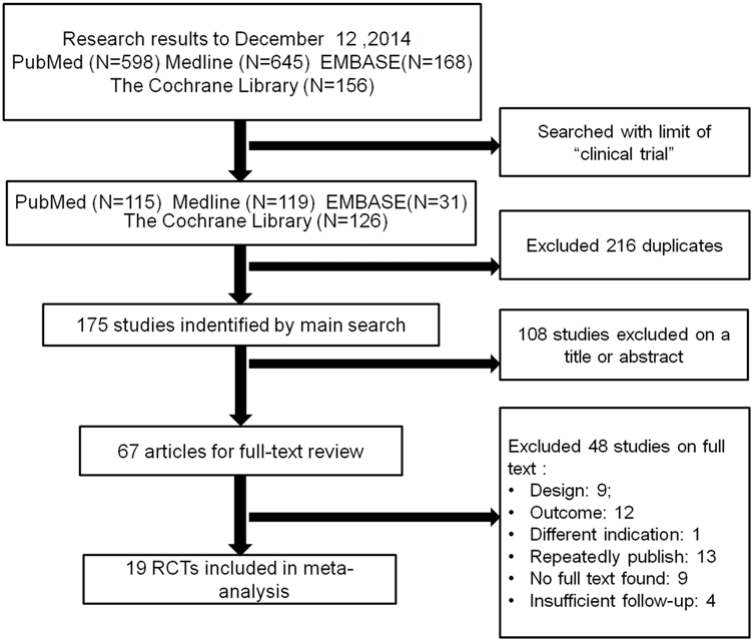
Flow chart for inclusion of studies.

As shown in Fig. 1, the literature search initially yielded 391 relevant trials from PubMed (*N* = 115), EMBASE (*N* = 31), Medline (*N* = 119), and the Cochrane Library (*N* = 126). After duplicates were excluded, 175 trials were retained. We (YJZ and CZL) reviewed the titles and abstracts of all 175 trials. Of those studies, 108 trials were excluded because they failed to meet the inclusion criteria. The remaining 67 studies underwent a full text review. Finally, nineteen RCTs with 4516 cases were included. The follow-up times were classified as short-term (2 or 3 years) or medium-term (4 or 5 years). We recorded the characteristics of the nineteen included trials (Table 1), as well as the details of the clinical outcome measurement (Table 2).

**Table 1:** Characteristics of the articles included in this review.

study	Sample size (CTDR/ACDF)	Mean age (years) (CTDR/ACDF)	Sex distribution (CTDR/ACDF) (male/female)	Intervention (CTDR/ACDF)
Heller et al.[38]	463(242/221)	44.4/ 44.7	(110/132)/(113/108)	Bryan/ ACDF with allograft and plate
Cheng. L et al. [39]	83(41/42)	47/47.7	(21/20)/(23/19)	Bryan/ ACDF with allograft and Orion System
Mummaneni et al.[40]	541(276/265)	43/43.9	(128/148)/(122/143)	Prestige ST/ACDF with allograft and Atlantis System
Coric et al. [41]	269(136/133)	43.7/43.9	(51/85)/(59/74)	Kineflex|C/ACDF with allograft and anterior plate
Phillips et al. [11]	342(189/153)	45.3/43.7	(113/105)/(96/89)	PCM/ ACDF with allograft and plate
Vaccaro et al. [42]	291(151/140)	43.4/44.4	(81/70)/(68/72)	SECURE-C/ACDF
Zigler et al. [43]	209(103/106)	42.1/43.5	(46/57)/(49/57)	ProDisc-C/ ACDF
Murrey et al. [30]	209(103/106)	42.1/43.5	(46/57)/(49/57)	ProDisc-C/ ACDF
Zhang. X et al. [44]	120(60/60)	44.8/45.6	(35/25)/(32/28)	Bryan/ ACDF with allograft and plate
Sasso et al. [17]	463(242/221)	44/44.7	(110/132)/(113/108)	Bryan/ACDF with allograft and plate
Riina et al. [29]	19(10/9)	41/38.1	(2/8)/(3/6)	Prestige ST/ACDF with allograft and Atlantis plate
Nabhan et al. [45]	49(25/24)	44	23/18	ProDisc-C/ACDF with Solis cage and plate
Burkus et al. [18]	541(276/265)	43.3/43.9	(128/148)/(122/143)	Prestige ST/ACDF with allograft and Atlantis System
Anakwenze et al. [10]	180(89/91)	42.2/41.7	(41/48)/(48/43)	ProDisc-C/ACDF
Coric et al. [19]	74(41/33)	49.5/49.3	(16/25)/(14/18)	Bryan, Kineflex|C/ ACDF using structural corticocancellous allograft and an anterior plate
CoriC et al. [46]	98(57/41)	46.6/46.3	(22/31)/(16/21)	Bryan, Kineflex|C or Discover/ ACDF with an allograft and anterior plate or artificial disc placement
Davis et al.[47]	330(225/105)	45.3/46.2	(113/112)/(45/60)	Mobi-C/ACDF
Davis et al.[48]	339(234/105)	NR	NR	Mobi-C/ACDF
Rozankovic et al.[49]	105(51/50)	41.32/41.94	(25/26)/(25/25)	Discover/ACDF

CTDR = cervical total disc replacement; ACDF = anterior cervical decompression and fusion; NR: not reported.

**Table 2:** Characteristics and clinical outcome measurements of the articles included in this review.

study	Follow-u (years)	Number of Cervical levels	Missing information (CTDR/ACDF)	US FDA IDE trial	Outcome measurement
Heller et al.[38]	2	1	12/27	YES	NDI, NDI success, pain assessment(NRS), SF-36, Neurological status, Reoperations, ROM, Complications, Overall success
Cheng. L et al. [39]	3	1,2 or3	0/2	NO	ROM, Complications
Mummaneni et al.[40]	2	1	53/67	YES	NDI, pain assessment(NRS), SF-36, Neurological status, ROM, Reoperations, Complications, Overall success
Coric et al. [41]	2	1	17/18	YES	Neurological status, Reoperations, Complications, Overall success
Phillips et al. [11]	2	1	23/34	YES	NDI success, Neurological status, Reoperations, ROM, Complications, Overall success
Vaccaro et al. [42]	2	1	49	YES	NDI success, Neurological status, Reoperations, Complications
Zigler et al. [43]	5	1	27/35	YES	Neurological status, Reoperations, Complications
Murrey et al. [30]	2	1	2/6	YES	NDI, NDI success, Neurological status, Reoperations, Complications, Overall success
Zhang. X et al. [44]	2	1	4/7	NO	NDI, ROM, pain assessment(VAS), Reoperations
Sasso et al. [17]	4	1	61/83	YES	NDI, NDI success, SF-36, pain assessment(NRS), Reoperations, Complications, Overall success
Riina et al. [29]	2	1	1/2	NO	NDI, SF-36, pain assessment(NRS), Neurological status
Nabhan et al. [45]	3	1	9	NO	pain assessment(VAS), Reoperations
Burkus et al. [18]	5	1	132/138	YES	NDI, SF-36, pain assessment(NRS), Neurological status, Reoperations
Anakwenze et al. [10]	2	1	NR	YES	ROM
Coric et al. [19]	4	1	11	YES	NDI success, Reoperations, Complications
CoriC et al. [46]	2	1 or 2	4/4	YES	Reoperations, Complications, Overall success
Davis et al.[47]	2	2	4/6	YES	Secondary surgical procedures, Neurological status, Adverse events
Davis et al.[48]	4	2	NR	YES	Secondary surgical procedures
Rozankovic et al.[49]	2	1	1/3	NO	NDI,VAS

CTDR = cervical total disc replacement; ACDF = anterior cervical decompression and fusion; NR: not reported

### Study Quality

**Fig 2:**
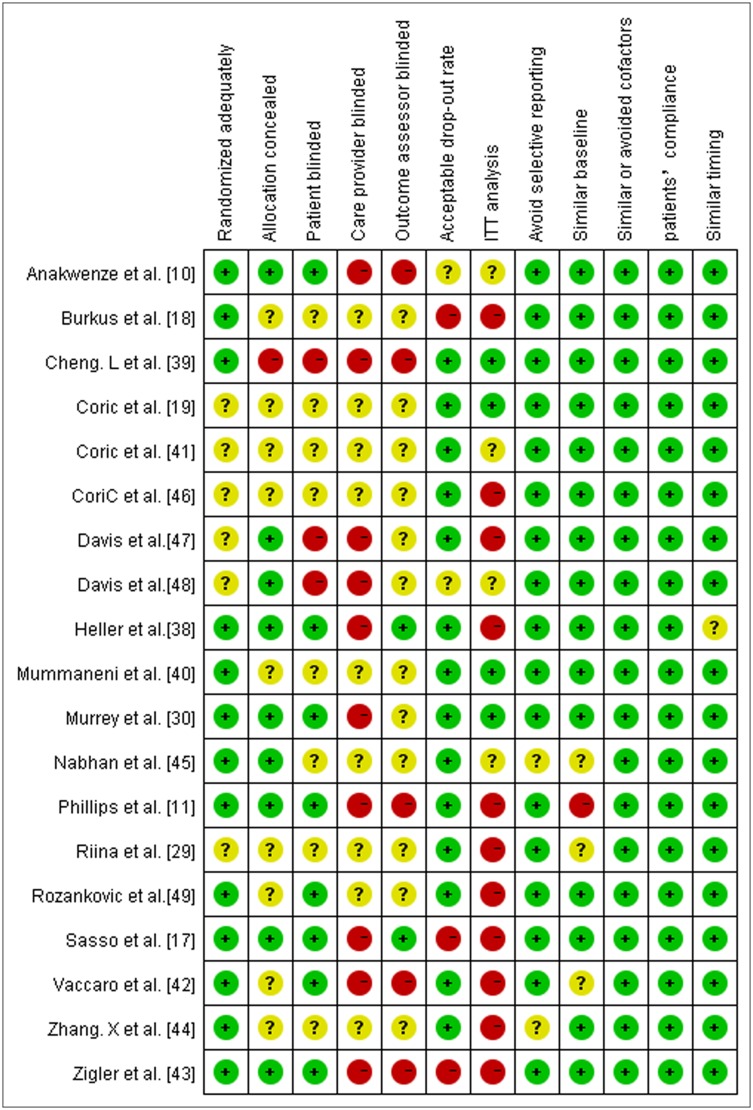
The risk of bias for the included studies was assessed in our meta-analysis.

According to the quality assessment criteria recommended by the Cochrane Back Review Group, eighteen studies with ‘‘low risk of bias” and one study with ‘‘high risk of bias” were found (Fig. 2). According to GRADE, a majority of the trials reviewed in our meta-analysis were moderate-quality studies (Tables 3 and 4).

**Table 3:** GRADE evidence profile of RCTs for compare CTDR and ACDF in short-term follow-up.

outcome	Number (treated/control)	Risk of bias*	Inconsistency^Ϯ^	Indirectness	Imprecision^ᵷ^	Publication bias	Quality
NDI	6(672/608)	serious	serious	no	no	undetected	Low
**NDI** ^‡^	5(621/558)	serious	no	no	no	undetected	Moderate
**NDI**(Bryan)	2(286/247)	serious	no	no	no	undetected	Moderate
**NDI**(Prestige-ST)	2(232/205)	serious	no	no	no	undetected	Moderate
NDI success	5(678/596)	serious	no	no	no	undetected	Moderate
Neurological success	8(1248/1018)	serious	no	no	no	undetected	Moderate
Arm pain							
NRS	3(492/421)	serious	no	no	no	undetected	Moderate
VAS	3(127/123)	serious	serious	no	serious	undetected	Very low
Neck pain							
NRS	3(492/421)	serious	no	no	no	undetected	Moderate
VAS	3(127/123)	serious	serious	no	serious	undetected	Very low
SF-36							
PCS	3(487/419)	serious	no	no	no	undetected	Moderate
MCS	2(257/225)	serious	no	no	no	undetected	Moderate
ROM							
Index	5(605/531)	serious	serious	no	serious	undetected	Very low
superior adjacent	2(330/312)	serious	no	no	serious	undetected	Low
inferior adjacent	2(193/166)	serious	no	no	serious	undetected	Low
Adverse event	8(1221/1012)	serious	no	no	no	undetected	Moderate
Secondary surgical procedures							
Index level	5(846/667)	serious	no	no	no	undetected	Moderate
Adjacent level	5(460/418)	serious	no	no	no	undetected	Moderate
Overall success	5(896/789)	serious	no	no	no	undetected	Moderate

GRADE = Grading of Recommendations Assessment, Development and Evaluation; CTDR = cervical total disc replacement; ACDF = anterior cervical decompression and fusion; NDI = neck disability index; NRS = numerical rating scale; VAS = visual analogue scale; PCS = physical component score; MCS = mental component score; ROM = range of motion.

* inadequate blinding, lack of allocation concealed in some trials may increase risk of bias;

Ϯ inconsistent report of outcomes and significant heterogeneity existed across the trials, but all were well explained by the subgroup analysis;

ᵷ if a study has a wide confidence interval around the estimate of the effect, or included patients less than 400, it may cause imprecision;

‡ NDI after sensitivity analysis;

**Table 4:** GRADE evidence profile of RCTs for compare CTDR and ACDF in midterm follow-up.

outcome	Number (treated/control)	Risk of bias*	Inconsistency^Ϯ^	Indirectness	Imprecision^ᵷ^	Publication bias	Quality
NDI	2(325/265)	serious	no	no	no	undetected	moderate
Neurological success	2(324/265)	serious	no	no	serious	undetected	low
NRS							
Neck pain	2(323/265)	serious	no	no	no	undetected	moderate
Arm pain	2(323/265)	serious	no	no	no	undetected	moderate
Secondary surgical procedures							
Index level	5(912/739)	serious	no	no	no	undetected	moderate
Adjacent level	5(912/739)	serious	no	no	no	undetected	moderate

GRADE = Grading of Recommendations Assessment, Development and Evaluation; CTDR = cervical total disc replacement; ACDF = anterior cervical decompression and fusion; NDI = neck disability index; NRS = numerical rating scale.

* inadequate blinding, lack of allocation concealed in some trials may increase risk of bias;

Ϯ inconsistent report of outcomes and significant heterogeneity existed across the trials, but all were well explained by the subgroup analysis;

ᵷ if a study has a wide confidence interval around the estimate of the effect, or included patients less than 400, it may cause imprecision;

### Neck Disability Index (NDI)

**Fig 3:**
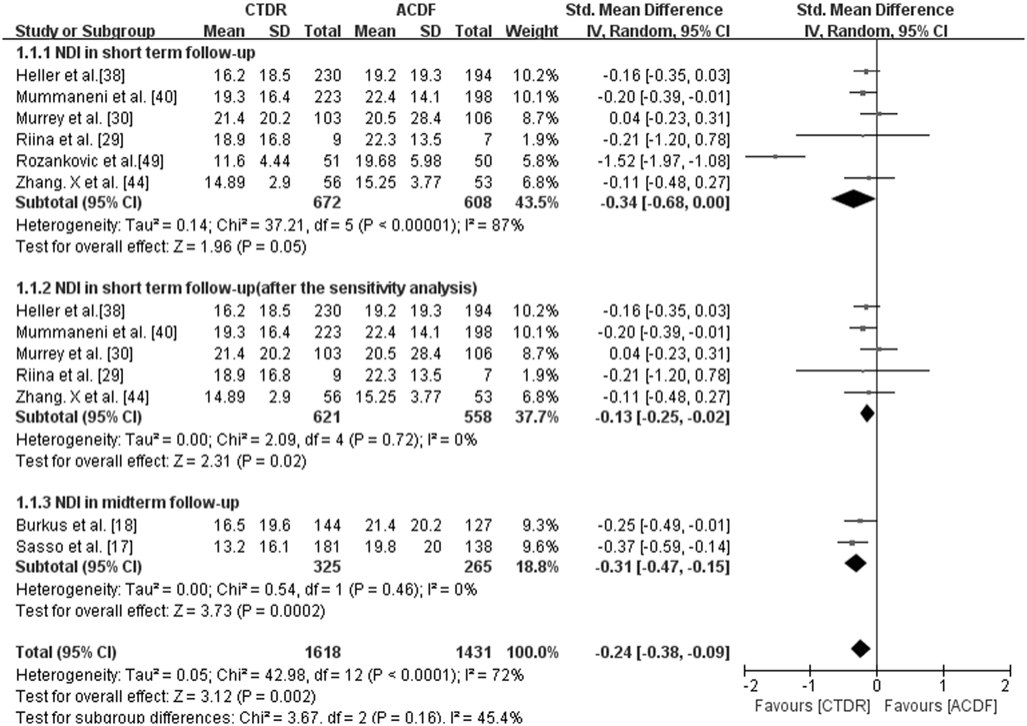
Comparison of NDI scores between the cervical total disc replacement (CTDR, experimental) group and the fusion (ACDF, control) group. IV = inverse variance, CI = confidence interval, and df = degrees of freedom.

With short-term follow-up, the CTDR group had statistically lower NDI scores (SMD, -0.34; 95% CI: -0.68 to 0.00, *P* = 0.05) than the ACDF group. However, there existed a substantial heterogeneity. Then we conducted sensitivity analysis and the result also showed that CTDR group had better NDI scores (SMD, -0.13; 95% CI: -0.25 to -0.02, *P* = 0.02) compared with ACDF group (Fig. 3). Additionally, we did subgroup analysis stratified by different types of prostheses. CTDR with Bryan had no significant difference compared with ACDF (SMD, -0.15; 95% CI: -0.32 to 0.02, P = 0.09). And CTDR with Prestige ST presented significantly lower NDI than ACDF (SMD, -0.20; 95% CI: -0.39 to -0.01, P = 0.04) (Table 5). With midterm follow-up, the NDI scores in the CTDR group were lower than those of the ACDF group (SMD, -0.31; 95% CI: -0.47 to -0.15, *P* = 0.0002) (Fig. 3).

**Table 5:** The pooled results of meta-analysis.

outcome	Sample size	Model	SWD (95%CI)	P_heterogeneity_	P-value	Favor
Short term						
NDI	1179	fixed	-0.13(-0.25– -0.02)	0.72	0.02	CTDR
NDI (Bryan)	533	fixed	-0.15(-0.32–0.02)	0.81	0.09	None
NDI (Prestige-ST)	437	fixed	-0.20(-0.39– -0.01)	0.99	0.04	CTDR
NRS (neck pain)	913	fixed	-0.14(-0.27– -0.01)	0.27	0.04	CTDR
NRS (arm pain)	913	fixed	-0.04(-0.17–0.09)	0.58	0.56	None
VAS (neck pain)	250	random	-1.28(-2.16– -0.40)	0.0001	0.004	CTDR
VAS (arm pain)	250	random	-1.03(-1.86– -0.19)	0.0002	0.02	CTDR
SF-36 (PCS)	906	fixed	-0.07(-0.20–0.06)	0.50	0.28	None
SF-36 (MCS)	482	fixed	0.05(-0.13–0.22)	0.32	0.62	None
ROM(index)	1136	random	-5.20(-6.77– -3.62)	<0.00001	<0.00001	CTDR
ROM(superior adjacent)	642	fixed	0.42(-0.28–1.12)	0.57	0.24	None
ROM(inferior adjacent)	359	fixed	-0.90(-1.84–0.04)	0.23	0.06	None
Medium-term						
NRS (neck pain)	588	fixed	-0.28(-0.44– -0.12)	0.33	0.0008	CTDR
NRS (arm pain)	588	fixed	-0.19(-0.35– -0.03)	0.70	0.02	CTDR

SWD = standardized mean difference; NDI = Neck Disability Index; NRS = numerical rating scale; VAS = visual analogue scale; PCS = physical component score; MCS = mental component score; ROM = range of motion. CTDR = cervical total disc replacement; None = no statistical differences;

### Neck Disability Index (NDI) success

**Fig 4:**
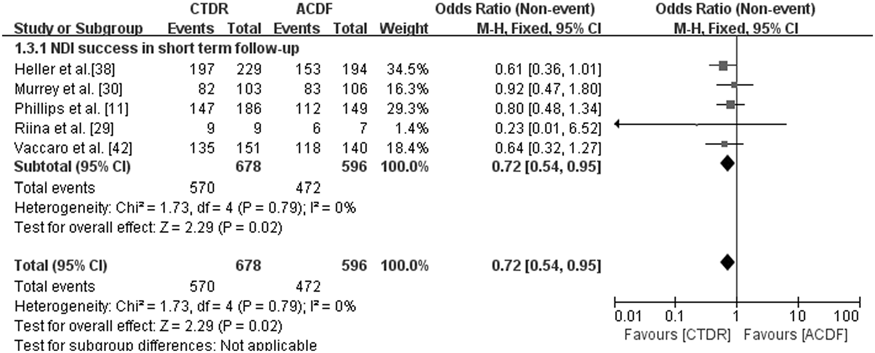
Comparison of NDI success between the cervical total disc replacement (CTDR, experimental) group and the fusion (ACDF, control) group in short-term follow-up. MH = Mantel-Haenszel, CI = confidence interval, and df = degrees of freedom.

NDI success was defined as a ≥15-point improvement in the NDI scores after surgery, which is generally regarded as a measure of function recovery [17]. Five studies with short-term follow-up provided NDI success data. As shown in Fig. 4, the CTDR group had a statistically higher NDI success rate than the ACDF group (OR, 0.72; 95% CI: 0.54 to 0.95, *P* = 0.02).

### Neurological success

**Fig 5:**
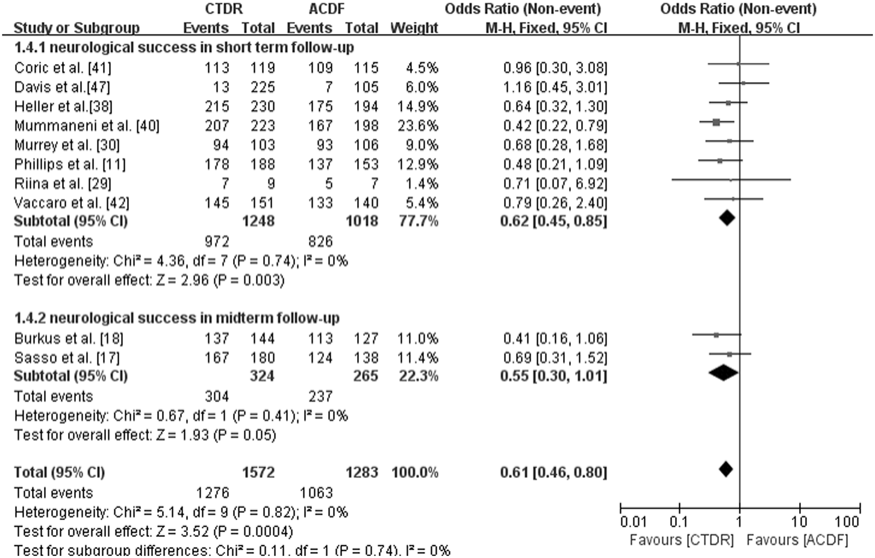
Comparison of Neurological success between the cervical total disc replacement (CTDR, experimental) group and the fusion (ACDF, control) group. MH = Mantel-Haenszel, CI = confidence interval, and df = degrees of freedom.

Maintenance or improvement of each neurological parameters (motor, sensory and reflexes) in standardized neurological examinations was interpreted as neurological success [11]. With short-term follow-up, there was a statistically higher neurological success rate in the CTDR group than in the ACDF group (OR, 0.62; 95% CI: 0.45 to 0.85, *P* = 0.003). With midterm follow-up, two studies provided neurological success data. Additionally, we found that more patients in the CTDR group achieved neurological success than in the ACDF group (OR, 0.55; 95% CI: 0.30 to 1.01, *P* = 0.05) (Fig. 5).

### Neck and arm pain

Neck and arm pain was measured using numerical rating scales or visual analog scales. Three studies with short-term follow up used numerical rating scales to measure neck and arm pain. Compared with the ACDF group, the CTDR group had significantly lower neck pain scores (SMD, -0.14; 95% CI: -0.27 to -0.01, *P* = 0.04). The arm pain scores did not differ significantly between the groups (SMD, -0.04; 95% CI: -0.17 to 0.09, *P* = 0.56). Three studies used visual analog scales to measure neck and arm pain. The CTDR group had significantly lower neck pain scores (SMD, -1.28; 95% CI: -2.16 to 0.40, *P* = 0.004) and significantly lower arm pain scores (SMD, -1.03; 95% CI: -1.86 to -0.19, *P* = 0.02) than the ACDF group. Two studies with midterm follow-up used NRS scores. The CTDR group presented significantly lower neck pain scores (SMD, -0.28; 95% CI: -0.44 to -0.12, *P* = 0.0008) and significantly lower arm pain scores (SMD, -0.19; 95% CI: -0.35 to -0.03, *P* = 0.02) (Table 5).

### SF-36

The SF-36 test is a self-administered questionnaire to assess general health status; it consists of a physical component summary (PCS) score and a mental component summary (MCS) score [17]. Three short-term follow-up studies provided PCS scores, and two of these studies provided MCS scores as well. As shown in table 5, no significant differences in the PCS scores (SMD, -0.07; 95% CI: -0.20 to 0.06, *P* = 0.28) and MCS scores (SMD, 0.05; 95% CI: -0.13 to 0.22, *P* = 0.62) were observed between the CTDR group and the ACDF group.

### Range of Motion (ROM)

The segmental motions were calculated from the angular motion on lateral flexion and extension radiographs of the cervical spine [18]. Five short-term follow-up studies provided ROM data at the index level. The CTDR group presented statistically better range of motion at the index level compared with the ACDF group (SMD, -5.20; 95% CI: -6.77 to -3.62, *P* < 0.00001). Two short-term follow-up studies provided ROM data at the adjacent level. No significant differences in ROM at the superior adjacent level (SMD, 0.42; 95% CI: -0.28 to 1.12, *P* = 0.24) or the inferior adjacent level (SMD, -0.90; 95% CI: -1.84 to 0.04, *P* = 0.06) were found (Table 5).

### Adverse events

**Fig 6:**
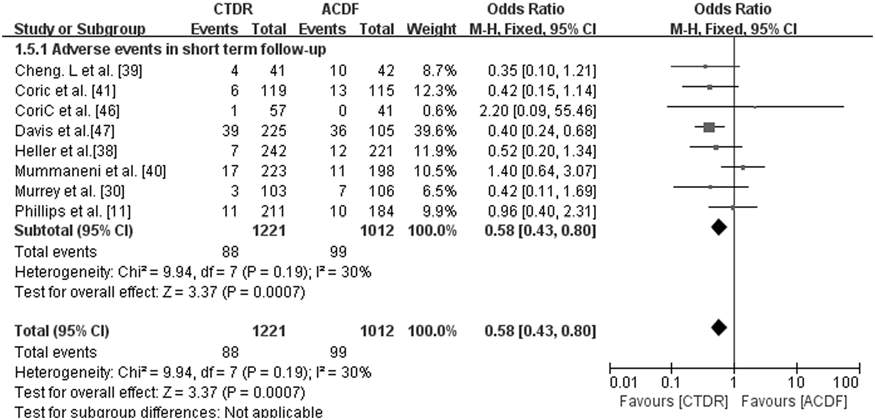
Comparison of Adverse events between the cervical total disc replacement (CTDR, experimental) group and the fusion (ACDF, control) group in short-term follow-up. MH = Mantel-Haenszel, CI = confidence interval, and df = degrees of freedom.

Eight studies that included a short-term follow-up investigated adverse events. We found that adverse events occurred more frequently in the ACDF group than in the CTDR group (OR, 0.58; 95% CI: 0.43 to 0.80, *P* = 0.0007) (Fig. 6). One study [19] with 74 patients had valid data for midterm follow-up.

### Secondary surgical procedures

**Fig 7:**
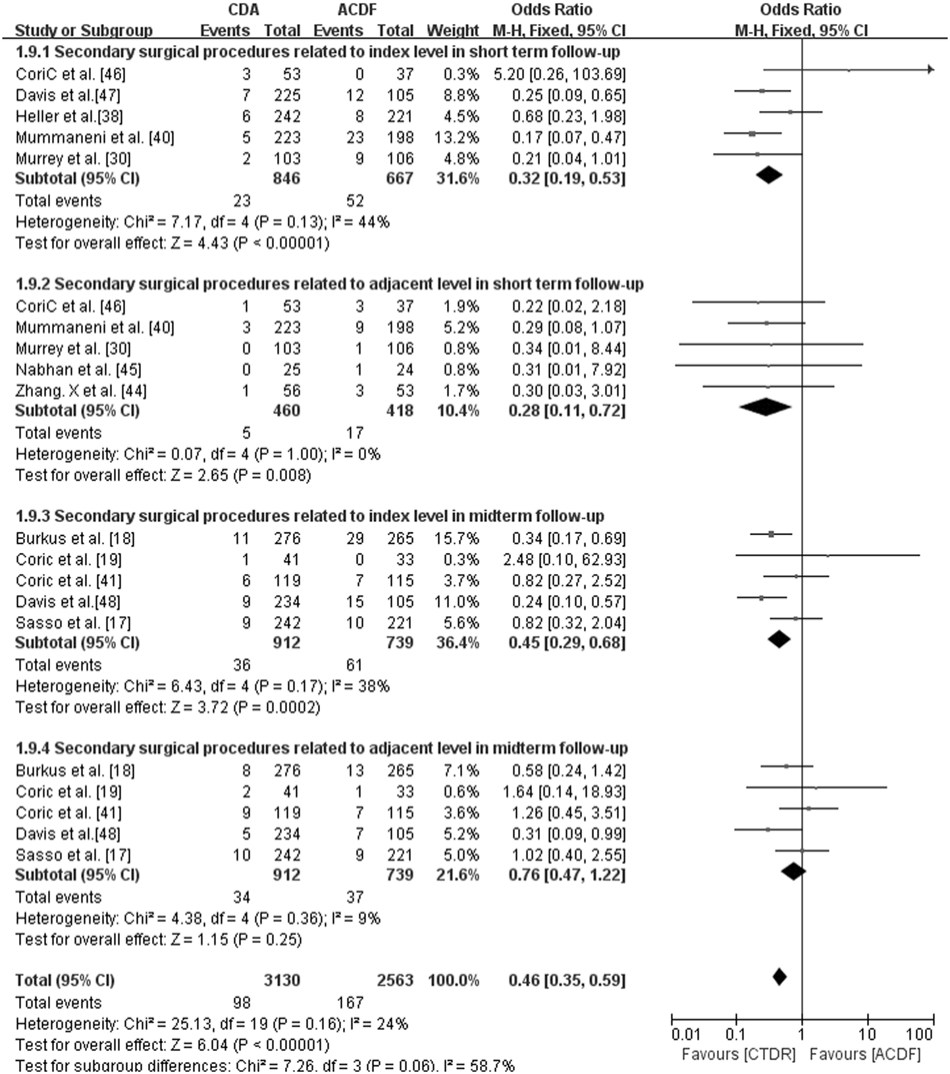
Comparison of Secondary surgical procedures between the cervical total disc replacement (CTDR, experimental) group and the fusion (ACDF, control) group. MH = Mantel-Haenszel, CI = confidence interval, and df = degrees of freedom.

Secondary surgical procedures were defined as any hardware removal, revisions, supplemental fixations, and reoperations [18]. They were typically used to resolve persistent neck or shoulder pain, dysphagia, prosthesis flexibility or adjacent level degeneration. For the short-term follow-up studies, we analyzed secondary surgical procedures at the index level and the adjacent level. We found that the CTDR group had significantly fewer secondary surgical procedures at the index (OR, 0.32; 95% CI: 0.19 to 0.53, P < 0.00001) and the adjacent level (OR, 0.28; 95% CI: 0.11 to 0.72, P = 0.008). For the studies with midterm follow-up, the rate of secondary surgical procedures at the adjacent level (OR, 0.76; 95% CI: 0.47 to 1.22, P = 0.25) was not significantly different between the groups. We noted significantly fewer secondary surgical procedures related to the index level in the CTDR group (OR, 0.45; 95% CI: 0.29 to 0.68, P = 0.0002) (Fig. 7).

### Overall Success

**Fig 8:**
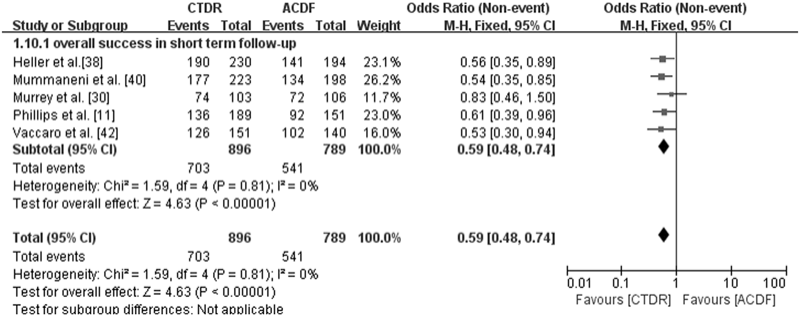
Comparison of overall success between the cervical total disc replacement (CTDR, experimental) group and the fusion (ACDF, control) group in short-term follow-up. MH = Mantel-Haenszel, CI = confidence interval, and df = degrees of freedom.

If a patient achieved all of the following items, the treatment was considered an overall success: NDI success, Neurological success, an absence of serious adverse events associated with the implant or procedure and without a secondary surgery or intervention [17]. Serious adverse events were defined as grade 3 or 4 adverse events based on the WHO criteria [20]. Six studies provided data on the overall success, and five of those had short-term follow-ups. One study with 463 patients focused on midterm follow-up [17]. The CTDR group presented a significantly higher overall success rate in studies with short-term (OR, 0.59; 95% CI: 0.48 to 0.74, *P* < 0.00001) and midterm follow-up (*P* = 0.004) (Fig. 8).

## Discussion

Most clinical data have supported CTDR as a viable alternative to ACDF. Several recent reports [13,14] have indicated that CTDR is not better than fusion in relieving symptoms associated with disc degeneration in the cervical spine. The comparison between CTDR and fusion was conducted in a few meta-analyses. With a total of eight RCTs, Yu et al. [21] reported CTDR was more effective than fusion in overall success rate and overall reoperation rate. Nevertheless, due to limited number of trials and the sample size, it was impractical to draw a conclusion that the patients with CTDR had better clinical status than those with fusion. Verma et al. [22] reported no significant difference in the rate of ASD between CTDR and fusion. However, this study only used the reoperation rate, without radiographical assessments, to evaluate the rate of ASD. It also might have a bias due to lower dropout rate in the CTDR group than fusion group. The meta-analysis conducted by Yin et al. [23] reported better function and lower complications in the patients with CTDR compared with the patients with fusion. Our results were consistent with this study, but they did not exclude the RCTs with one year follow-up. And more stringent scores, such as overall success, were not utilized in their study. Overall, the efficacy and safety of CTDR procedures are still controversial. Moreover, most relevant meta-analyses [14,21–23] only chose the RCTs published before 2012, whereas several latest RCTs were reported in the last two years. We performed a meta-analysis of nineteen RCTs to determine whether CTDR was superior to ACDF.

This meta-analysis showed that patients treated with CTDR had better NDI improvement and higher NDI success rates than those treated with ACDF in short-term and midterm follow-up. However, in the subgroup analysis of NDI, CTDR with Bryan had no significant difference compared with ACDF, while CTDR with Prestige ST presented significantly lower NDI than ACDF. The result indicated that different types of prosthesis might have different efficacy and safety. Due to limited number of included articles, the other outcomes cannot be performed subgroup analyses stratified by types of prostheses. In addition to superior NDI outcomes, higher neurological success rates were reported in the CTDR group than in the fusion group. Regarding pain relief, we found that the CTDR group had lower neck pain (NRS) scores in short-term follow-up and lower neck and arm pain (NRS) scores in the midterm follow-up. Additionally, the neck and arm pain (VAS) scores in short-term follow-up also demonstrated the CTDR group had a favorable outcome. Overall, the CTDR group showed better functional improvement than the fusion group.

Compared with fusion, CTDR resulted in better segmental motion at the index level, which was consistent with the results of previous studies [24,25]. As demonstrated in previous studies, impaired ROM at the index level was normally in compensation at the adjacent levels in spinal procedures. Worse segmental motion at the index level would result in a higher load and intradiscal pressure on the adjacent segments, which would accelerate the degeneration of the adjacent segments. Eck JC et al. [7] explained that high intradiscal pressure led to an accumulation of waste products in the disc, which could cause cell death and disrupt metabolism. To avoid detrimental non-physiological loading exertion on the adjacent segments, researchers have focused their attention on developing CTDR to maintain the basic motion of intervertebral segments [26]. The assumption that adjacent segment disease arises from spinal fusion with iatrogenic motion restriction is under debate. Some investigators have hypothesized that adjacent segment disease signifies progression of the natural history of spinal segmental degeneration [26,27]. The pooled results of this analysis indicated that although CTDR could retain segmental motion at the index level more effectively than fusion, the ROMs at the superior adjacent and inferior adjacent level were not statistically different. On the other hand, our data indicated that the CTDR group had significantly fewer secondary surgical procedures attributable to adjacent segment degeneration in the short-term follow-up. Hence, we hypothesized that some other factors influenced the incidence of adjacent segment degeneration. Nunley and colleagues [26] considered that bone mineral density and presence of concurrent lumbar degeneration had a significant effect in the incidence of adjacent segment degeneration.

In addition to fewer secondary surgical procedures, the CTDR group also had fewer adverse events. The most frequent adverse events of CTDR include heterotopic ossification, segmental kyphosis, migration, or subsidence of the artificial disc. Previous generations of CTDR were typically associated with high adverse events rates. For instance, the Bristol–Cummins joint was very efficient in maintaining motion; however, it is always complicated by joint subluxation, screw failure and high rates of dysphagia [28]. As technology has progressed, increasing numbers of new models have been developed, including the Bryan, Prestige ST, and ProDisc-C. The Bryan prosthesis consists of a polyurethane nucleus in a saline solution bath sandwiched between two titanium alloy surfaces. And the device allows for bone ingrowth from the vertebral end plates [17]. The Prestige ST prosthesis is a dynamic stainless steel device that consists of two metal plates, and the device permits segmental spinal motion through a ball-and-trough mechanism and maintains disc space height [29]. The ProDisc-C prosthesis is composed of two cobalt chromium molybdenum alloy end plates with midline keels and an ultrahigh–molecular weight polyethylene (UHMWPE) inlay. The midline keel on the vertebral surfaces provides fixation while a plasma-spray titanium coating encourages bony on-growth for longer term stability [30]. Those prostheses have advantages over previous models in retaining segmental motion, disc height and lordosis. Additionally, they have longer-term durability and produce less inflammatory reaction or osteolysis. Therefore, they can better mimic a natural intervertebral disc and, consequently, can obtain better clinical outcomes with fewer prosthesis-related complications [12]. For fusion surgery, the most frequent adverse events include dysphagia, dural injury, hoarseness, worsening of neurological symptoms, and graft extrusion. Dysphagia was reported to be the most common complication, occurring in 3.3% of patients, whereas the overall ACDF-related complication rate was 8.4% [5]. CTDR could substantially decrease the incidence of dysphagia to a greater degree than fusion because the CTDR procedure demands less esophageal retraction and consequently reduces the intraesophageal pressure [31].

Overall success is measured by a composite score that includes NDI success, neurological status, adverse events, and subsequent surgery. The composite definition of success was significantly more stringent than the traditional definition because the procedure would be considered a failure even if a patient failed only one component [32]. In this meta-analysis, the CTDR group had a higher overall success rate than the fusion group. We hypothesized that the unique advantages of the prostheses might explain the better overall success in the CTDR group than in the ACDF group. Additional effort is required to develop novel prostheses that mimic natural intervertebral discs more closely. Innovative 3D-printing technology may provide a possible solution for this issue [33–35].

Recently, several non-RCTs were published to evaluate the long-term efficacy and safety of CTDR. Malham et al. [36] reported CTDR had a pretty good improvement in functional outcomes, as well as 37% of heterotopic ossification and 21% of radiographic adjacent segment disease, with average follow-up of 7.7 years. Zhang et al. [37] also reported CTDR had satisfactory functional outcomes without any significant complication. In this study, mean ROM at index levels is 8.6°±0.2°and 81.3% of the segments were mobile at 6 years, which indicated that ROM was preserved at index levels. In a word, the long-term studies displayed favorable efficacy and safety of CTDR, which was consistent with our meta-analysis.

The validity of the study results was limited by several factors. First, several studies presented low quality evidence resulting from inadequate blinding, insufficient allocation concealment, and imprecision. Second, missing information such as the absence of ITT analysis and loss to follow-up led to incomplete data and potentially biased results. Third, a non-inferiority study design was utilized in almost all the RCTs, and this design is typically less stringent in demonstrating efficacy than standard clinical trials. Fourth, we did not assess publication bias because of the small sample sizes, which led to imprecision across the studies. Fifth, no data on long-term efficacy and safety were available because the follow-up was no more than five years in all the included studies.

This meta-analysis indicated that in efficacy and safety, CTDR was superior to ACDF. Longer-term, multicenter studies are required for a better evaluation of the long-term efficacy and safety of these two procedures.

## Supporting Information

S1 Table Excluded Papers. (DOCX)

S1 PRISMA Checklist (DOC)
