# Supplementary material for: Cervical Total Disc Replacement is Superior to Anterior Cervical Decompression and Fusion: A Meta-Analysis of Prospective Randomized Controlled Trials
Source: PLoS One. 2015 Mar 30;10(3):e0117826. doi: 10.1371/journal.pone.0117826 (PMC4379027; doi:10.1371/journal.pone.0117826)
Supplement: S1 Table — (DOCX) [file pone.0117826.s001.docx]

**Table S****1 Excluded Papers**

| First Author | Year | Journal | Exclusion Reason |
| --- | --- | --- | --- |
| Anderson PA | 2008 | Spine (Phila Pa 1976) | Repeated report |
| Attabib N | 2008 | Canadian journal of surgery Journal canadien de chirurgie | No full text found |
| Auerbach JD | 2011 | Spine (Phila Pa 1976) | Outcome |
| Bae HW | 2010 | Spine journal | No full text found |
| Bartels RH | 2006 | BMC Musculoskelet Disord | Outcome |
| Beaurain J | 2009 | Eur Spine J | Study design |
| Bhadra AK | 2009 | Eur Spine J | Study design |
| Cao J | 2008 | Chinese journal of reparative and reconstructive surgery | No full text found |
| Chen Y | 2013 | Orthopedics | Target population is different |
| Cheng L | 2009 | Int Orthop | Repeated report |
| Coric D | 2006 | J Neurosurg Spine | Insufficient Follow-up |
| Davis R | 2011 | Eur Spine J | No full text found |
| Davis R | 2012 | confernce | No full text found |
| Delamarter RB | 2010 | SAS Journal | Repeated report |
| Delamarter RB | 2013 | Spine | Repeated report |
| Garrido BJ | 2010 | J Spinal Disord Tech | Repeated report |
| Garrido BJ, | 2011 | Journal of Bone and Joint Surgery American Volume | Outcome |
| Guyer RD | 2010 | Spine journal | No full text found |
| Jawahar A | 2010 | Spine J | Outcome |
| Kelly MP | 2009 | 37th Annual Meeting of the Cervical Spine Research Society, | No full text found |
| Kelly MP | 2011 | Spine (Phila Pa 1976) | Repeated report |
| Li Z | 2014 | Journal of clinical neuroscience | No CTDR |
| Maldonado CV | 2011 | Eur Spine J | Study design |
| Marzluff J | 2010 | Spine journal | No full text found |
| McAfee PC | 2010 | J Spinal Disord Tech | Outcome |
| McDonald CP | 2014 | J Neurosurg Spine | Study design |
| Murrey DB | 2008 | SAS journal | Outcome |
| Nabhan A | 2007 | Eur Spine J | Insufficient Follow-up |
| Nabhan A | 2006 | Eur Spine J | Repeated report |
| Nunley PD | 2012 | Spine (Phila Pa 1976) | Outcome |
| Park DK | 2011 | Spine (Phila Pa 1976) | Insufficient Follow-up |
| Park JH | 2008 | Journal of Korean Neurosurgical Society | Study design |
| Peng-Fei S | 2008 | Int Orthop | Insufficient Follow-up |
| Porchet F | 2004 | Neurosurg Focus | Outcome |
| Powell JW | 2010 | J Spinal Disord Tech | Repeated report |
| Robertson JT | 2005 | J Neurosurg Spine | Study design |
| Rohl K | 2009 | Spinal Cord | Outcome |
| Sasso RC | 2008 | J Spinal Disord Tech | Repeated report |
| Sasso RC | 2008 | J Spinal Disord Tech | Repeated report |
| Sasso RC | 2007 | Spine (Phila Pa 1976) | Repeated report |
| Sasso RC, | 2007 | J Spinal Disord Tech | Repeated report |
| Segebarth B | 2010 | SAS journal | Outcome |
| Skeppholm M | 2013 | Spine (Phila Pa 1976) | Outcome |
| Steinmetz MP | 2008 | Neurosurgery | Repeated report |
| Wang Y | 2008 | Zhonghua Wai Ke Za Zhi | No full text found |
| Yu S | 2012 | Chinese Medical Journal | Study design |
| Zhang HX | 2014 | Int Orthop | Outcome |
| L.-Y. Fay | 2013 | Eur Spine J | Study design |
